# Training residents in patient-centred communication and empathy: evaluation from patients, observers and residents

**DOI:** 10.1186/s12909-019-1555-5

**Published:** 2019-05-02

**Authors:** J. Noordman, B. Post, A. A. M. van Dartel, J. M. A. Slits, T. C. olde Hartman

**Affiliations:** 1 Department of Primary and Community care, Radboud Institute for Health Sciences, Radboud University Medical Center, Nijmegen, The Netherlands; 2 Nivel, Netherlands institute for health services research, Utrecht, The Netherlands; 3 Department of Neurology, Donders Institute for Brain, Cognition and Behaviour, Radboud University Medical Center, Nijmegen, The Netherlands; 4 Department of Primary and Community care, Donders Institute for Brain, Cognition and Behaviour, Radboud University Medical Center, Nijmegen, The Netherlands

**Keywords:** Communication, Residents, Patients, Empathy, Patient-centred, Computer use, Video recordings, Questionnaires, Interviews

## Abstract

**Background:**

Patient-centred communication and empathy are key enablers for patient-centred care. However, several studies suggest a downward trend regarding the empathic communication skills of physicians during medical residency. It is known that communication training can have a positive effect on patient-centred communication, empathy and relational skills. Training residents in patient-centred communication and empathy can be an opportunity to improve the patient-centred care. To evaluate the training a tri-focal perspective will be used.

**Methods:**

A 3-day training was developed to improve residents’ patient-centred communication and empathy skills at an academic medical health centre, in the Netherlands. The training included: (1) the basics of patient-centred communication and empathy (through presentations, scientific literature), (2) practicing with actors, and (3) reflecting on residents’ video recorded consultations (by themselves and communication experts). A pilot study with a pre-post design was conducted to evaluate the training from patient and observer perspectives. Semi-structured interviews were used to get insight into residents’ perspective. Nine residents from different specialities followed the training and enrolled in the pilot study. During two random days consultations between residents and patients were video recorded. Patients were asked to fill in two questionnaires, indicating their perspective on residents’ empathy and communication skills before as well as after the consultation. All video recorded consultations were coded to rate residents’ communication skills, empathy, computer use and agenda-setting. Statistical analysis were performed using multilevel analysis.

**Results:**

A total of 137 eligible patients took part in the pilot study. Trained residents showed significant improvement in patient-rated empathy scores. According to observers, residents’ computer use improved significantly after the training. The communication skills of trained residents did not improve significantly. Agenda setting by residents showed a downward trend. Almost all residents were satisfied with the training, especially with the video-feedback.

**Conclusions:**

A brief training significantly increased residents’ empathy scores according to patients and significantly decreased residents’ computer use according to observers. These findings indicate that the quality of patient-centred care can be improved by integrating patient-centred communication into residency programs, at an academic medical health centre. The ultimate goal is to structurally embed the training in residents’ education program.

## Background

Delivering high quality, patient-centred care is an important goal in medical practice. The concept of patient-centred care contains paying more attention to patients’ view on care, promoting involvement of patients in their own healthcare, in order for patients to have increased agency over their own health [1, 2]. More than 75% of patients prefer a patient-centred approach [3]. Many different definitions of the concept of patient-centred care have been proposed, but all include the key enablers of patient-centred communication, as well as empathy of the physician as an essential characteristic [1, 2, 4–6]. According to Brouwers and colleagues [5], based on the definition by Stewart et al. [1], ‘patient-centred communication ideally includes 6 dimensions: exploring both the disease and the illness experience; understanding the whole person; finding common ground between the physician and patient; incorporating prevention and health promotion; enhancing the doctor–patient relationship, and ‘being realistic’ about personal limitations and issues such as the availability of time and resources’. Empathy can be defined as ‘the ability to understand patients’ situation, perspective and feelings, and to communicate that understanding to the patient’ [7]. Previously, patient-centred communication studies have shown positive outcomes on patients’ satisfaction, adherence and several (mental) health outcomes (e.g. [8]). In addition, several studies into empathic communication show an increase of patient satisfaction, improved adherence to therapy, better patients’ health outcomes, decreased physicians’ burn-out and increased physician well-being [9–12]. For example, a randomized controlled trial studied the effect of physician-patient interaction on the duration of common cold. They concluded that positive patient perception of practitioner’s empathy is significantly correlated to the duration and severity of common-cold-symptoms [11]. Furthermore, another study found that the empathic capacity of doctors (rated by the doctors themselves) showed a positive correlation between doctors’ empathy scores and glycated hemoglobin control of diabetes patients [9].

Despite these positive influences of empathy, there are studies suggesting a downward trend regarding empathic communication skills of physicians during medical residency [4, 12–14]. A longitudinal study showed a significant decline in mean empathy scores in the third year of medical school, compared to first year students [9]. This decline continues during residency training [4]. The erosion of empathy can be attributed to several factors, including lack of role models, fear of making mistakes, sleep loss, a high volume of materials to learn, time pressure and patient and environmental factors [14].

Training residents in both patient-centred communication and empathy seems to be an opportunity to improve the patient-centred care. It is known that a communication training can have a positive effect on patient-centred communication, empathy and relational skills [5, 15, 16]. Previous research among general practitioners (GPs) [17] and practice nurses [18], showed significant improvements on patient-centred communication skills using video feedback. Therefore, a 3 = three day training was designed to improve the communication skills of residents by focusing on the basics of patient-centred communication and empathy, including practicing with actors and reflecting on residents’ own video recorded consultations. We hypothesized that a training in patient-centred communication and empathy for residents would increase residents’ empathy and thereby improve the quality of patient-centred care, at an academic medical health centre in the Netherlands. To evaluate the training a tri-focal perspective will be used.

## Methods

### Aim

The aim of this study was to evaluate residents’ training ‘patient-centred communication and empathy’ from the perspective of patients, observers and residents themselves.

### Design

A pilot study with a pretest-posttest design was conducted to evaluate the training ‘patient-centred communication and empathy’, from patients’ and observers’ perspective. In addition, semi-structured interviews were used to gain insight into residents’ perspective on the training.

### Training ‘patient-centred communication and empathy’

A 3 = three day training ‘patient-centred communication and empathy’ was set up to improve the communication skills of residents, at an academic medical health centre in the Netherlands. The training protocol was developed by a GP with interest in patient-centred communication and empathy, and a neurologist with affinity with empathy, education and medical specialty training, in cooperation with several experts in patient-centred communication and empathy.

The training included: (1) the basics of patient-centred communication and empathy (through presentations and scientific literature, e.g. the following literature was included [19, 20], among others), (2) practicing (difficult) situations with actors (i.e. simulation patients) based on residents’ own experiences (e.g. coping with ‘a demanding patient’), (3) intervision with colleague residents, and (4) reflecting on residents’ video recorded consultations (by themselves and by experts; both individual and group feedback).

The training consisted of three monthly sessions: two whole-day sessions and one half-day session, between April and June 2017 at an academic medical health centre in the Netherlands. The sessions highlighted different aspects. The first session drew attention to the performance of a good consultation. In the second session, residents were taught about personalised communication and empathy and its effectiveness on health outcomes, and the third session was developed to sustain the acquired skills. To prepare for these sessions, the residents had to fulfil short assignments, i.e. sharing their motivation and personal learning goals, reading literature, video record one or two of their own consultation(s), reflect on their own communication and evaluate their learning goals (i.e. self-directed learning). Two months before the training, residents were asked to hand in three of their own video recorded consultations. An independent GP observed the videos and provided individualized video feedback based on the “Maastrichtse Anamnese en Advies Scorelijst” (MAAS-global) [21]. The three self-selected video recordings of residents were part of the training for residents, but were not included in the pilot study to avoid bias. Based on these videos the trainers decided to emphasise ‘agenda setting’ and ‘computer use’ during the training.

### Participants and procedure

From March to October 2016, we enrolled residents from three specialties at the academic medical health centre, who were given written information about the study.

A resident in the Netherlands is a doctor with a basic medical degree who is in training for a medical specialty. Residents were eligible if they (1) were currently in training, (2) had completed their first internship at the outpatient clinic, and (3) had clinical interactions with adult outpatients. Residents rotating outside this academic medical health centre during the training period were excluded. Participation was voluntary. Our aim was to include 10 residents.

All adult and proficient Dutch-speaking patients who consulted the resident physician at the outpatient clinic were asked by a research assistant to participate in the study. The research assistant explained the purpose of the study, what was expected of patients who participated, and provided written information about the study. Subsequently, patients gave their written informed consent.

The patients’ visit to the resident physician was recorded using an unmanned digital camera. Participating patients were asked to fill in both a pre- and post-consultation questionnaire. In case of a patient willing to fill in the pre- and post-consultation questionnaire but refusing the consultation being video recorded, this was noted on the informed consent form and the digital camera was switched off. To maintain the pre-post nature of the study, different patients completed the questionnaires pre- and post-intervention.

An overview of the included evaluation measures from patient, observer and resident perspectives are presented in Table 1.

**Table 1:** Measures to evaluate the training from patient, observer and resident perspectives

	Pre-intervention	Post-intervention
Patient perspective	- Importance and residents’ performance scores of consultation aspects (QUOTE-COMM) -State anxiety (STAI) -Residents’ empathy and relational skills (CARE)	- Importance and residents’ performance scores of consultation aspects (QUOTE-COMM) -State anxiety (STAI) -Residents’ empathy and relational skills (CARE)
Observer perspective	- Communication skills and empathy (MAAS-global) - Agenda setting (present or not) - Computer use (self-developed protocol)	- Communication skills and empathy (MAAS-global) - Agenda setting (present or not) - Computer use (self-developed protocol)
Resident perspective	n.a.	Expectations and learning goals Positive and improvement point Future of training and education

### Measures from patients’ perspective

In the pre-consultation questionnaire patients’ socio-demographic characteristics, patients’ expectations (also referred to as importance score) about the consultation and state anxiety were obtained. In the post-consultation questionnaire, fulfilment of patients’ pre-consultation expectations (also referred to as performance score), state anxiety and the residents’ empathy and relational skills from patients’ perspective were measured.

### Socio-demographic characteristics

Measured aspects were patients’ year of birth, sex, educational level, number of hours of work per week, familiarity with the resident physician and the most important reason(s) for encounter with the resident physician.

### Importance and residents’ performance scores of consultation aspects

The Quality of Communication Through Patient’ Eyes (QUOTE COMM) [22] was used to assess the importance and residents’ performance score of several consultation aspects. This questionnaire consists of 13 items, divided into two categories: an affect-oriented scale of seven communication aspects and a task-oriented scale of six communication aspects. Before the consultation, patients rated the importance on various aspects (for example: I find it important that the doctor explains well what’s wrong) on a four-point Likert-type scale (1, not important to 4, extremely important). After the consultation, patients rated the residents’ performance score on the same aspects (for example: the doctor explained well what’s wrong) on a four-point Likert scale (1, not performed to 4, yes). Furthermore, a therapy subscale of six questions was added; two about the proposed therapy and four about reaching agreement on the intended treatment. Importance and residents’ performance score were defined as the absolute score on the Likert scale. The scores were dichotomized in the analysis.

### State anxiety

State anxiety refers to a temporary condition in response to some perceived threat. In this study, a threat could be a distressing consultation with the resident physician. To measure state anxiety, the Spielberger State-Trait Anxiety Inventory (STAI) was used before and after the consultation with the resident physician. The STAI, consisting of two scales, is a widely used self-report scale to measure anxiety. Each scale contains 20 items and each item is rated on a four-point ordinal scale (1, not at all to 4, very). The STAI has been translated to Dutch and validation has proven its reliability and sensitivity in the measure of anxiety [23]. In this study, we used a short version of the Dutch translation of the STAI. The short version contains 10 items and has good content validity and can be used adequately to assess trait anxiety. Questionnaires whereby less than 70% of the STAI questions were answered, were excluded. Subsequently, the anxiety level before and after consultation was calculated by averaging the scores.

### Residents’ empathy and relational skills

The Consultation and Relational Empathy Measure (CARE) is a 10-item patient-rated questionnaire for assessing empathy in the doctor-patient consultation [24]. Resident physicians who are given a high score are experienced as more empathic by their patient than resident physicians who are given a low score. A Dutch version of the CARE has been made and validity and reliability has been tested [25]. The total CARE measure score was calculated by including only those questionnaires which had no missing values or containing one or two ‘not applicable’ responses or missing values (or one of each). In that case, an overall score was calculated by replacing missing values by the mean calculated value from the valid items scores obtained for the same case. Questionnaires were excluded if less than 80% of the CARE questions were answered.

### Measures from observers’ perspective

The video recorded consultations were scored by an observer (JS), who was trained by an experienced researcher skilled in communication aspects (JN), to rate residents’ communication skills, including level of empathy, their computer use and agenda setting during the consultations. For inter-rater reliability, 10 % of the video recorded consultations were independently scored by the experienced observer (JN).

### Communication skills and empathy

The “Maastrichtse Anamnese en Advies Scorelijst” (MAAS-Global) [21] includes seventeen items regarding physician-patient communication which are subdivided in phase-specific communication skills (from ‘introduction’ to ‘evaluation’ of the consultation), general communication skills (e.g. exploration, structuring, empathy) and content aspects (e.g. anamnesis). We did not observe the content aspects in this study since we focus on communication skills. Therefore, we scored the first thirteen items (see Table 5). Each item is scored on a seven-point rating scale, ranging from 0 (‘not present’) to 6 (‘excellent’). The total score is the average of all scored items, as the two items ‘follow-up consultation’ and ‘physical examination’ may be inapplicable. The validity and reliability of the MAAS-Global were found to be satisfactory in previous studies [26–28].

### Agenda setting

The aspects agenda setting and computer use were highlighted during the training program and therefore added to the observation protocol. Agenda setting was scored as ‘present’ or ‘absent’ in the beginning of the consultation.

### Computer use

To score computer use during residents’ consultations, the video recorded consultations were reviewed by an observer using a previous developed observation list [29]. For each consultation, the observer described when and how the resident used the computer. There are two categories: ‘no computer use’ and ‘computer use’. If computer use is present, seven categories of computer use were defined. The computer may be used: (a) to search for or read something (e.g. during history taking); (b) to prescribe medication or refer a patient; (c) while the patient is changing clothes (for physical examination); (d) while the resident is talking; (e) while the patient is talking; (f) while the patient waits silently and (g) for other purposes (e.g. to make an appointment). As a total score of computer use, the observer noted the degree of intrusion of computer use ranging from 0 (‘not present’) to 5 (‘very annoying’). Computer use was considered annoying in case of: using the computer while the patient was talking or while residents explained something to the patient or asked a (personal) question. Activities to make computer use less annoying were: mentioning the computer use by the resident and computer use supporting the consultation (i.e. display diagnostic tests and search for information needed at that time).

### Measures from residents’ perspective

After the training, participating residents were interviewed to evaluate the training. This semi-structured in-depth interview included the following topics: (1) expectations and learning goals of residents, (2) positive and improvement points and, (3) the future of the training and education for residents. The interviews were audio-recorded, transcribed verbatim and analysed on content.

### Statistical analysis

Differences between patient characteristics before and after the intervention were investigated using independent t-tests for continuous variables and chi-square tests for dichotomous variables.

Multilevel analysis was used to account for clustering of patients (level 1) nested within residents (level 2), with a random intercept model or logistic regression for either scale variables or dichotomous variables. A *p*-value of less than 0.05 was considered statistically significant. To indicate inter-rater reliability for the coded video recorded consultations, Cohen’s kappa was used [30]. Statistical analysis was performed using Statistical Package for the Social Sciences (SPSS) version 22 (SPSS Inc., Chicago, Illinois, USA).

## Results

### Participants

Of 14 initially interested residents, 10 (71%) agreed to participate. However, due to ending her proceedings in the academic medical health centre, one resident did not participate in the training program. Therefore, our study consisted of nine residents, in internal medicine (*n* = 1), neurology (*n* = 4) and oral and maxillofacial surgery (n = 4). The majority of them were women (77.8%) with a mean age of 30.4 years (SD 2.6). The participating residents worked on average 44.8 h (SD 3.7) and had 3.6 years (SD 2.6) experience as a resident. Of the nine residents, seven were able to finish the entire program. Two residents withdrew due to proceedings abroad and private circumstances. This resulted in the participation of nine residents pre-intervention and seven post-intervention.

**Fig. 1:**
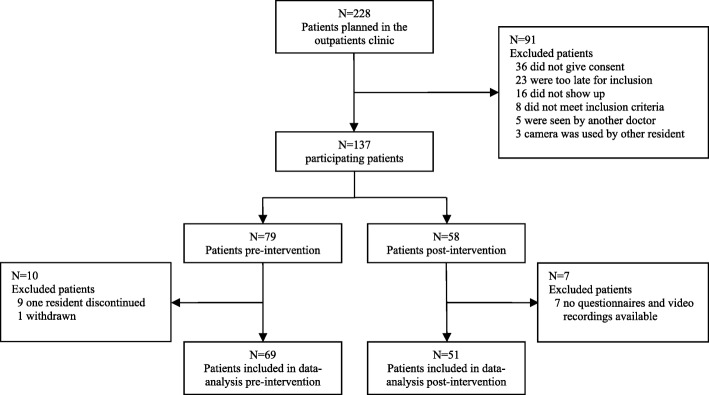
Study recruitment flow

A total of 228 patients, who were visiting the outpatient clinic to consult the participating residents, were approached to participate in the study. Of these, 91 were not able to participate for several reasons. Of 137 participating patients, 17 were not included in the analysis as one resident stopped working at the academic medical health centre and withdrew from participating, one patient withdrew from participating and of several patients we only received the socio-demographic information. Figure 1 shows the recruitment flow and Table 2 shows the comparison of socio-demographic characteristics of the participating patients before and after the intervention. There was no significant difference in patient characteristics before and after the intervention.

**Table 2:** Comparison of patients' characteristics pre- and post-intervention

	Pre-intervention (*N* = 69)	Post-intervention (*N* = 51)	*p* value
Mean age in years (SD)	56.1 (15.5)	51.9 (18.2)	0.17
Men (%)	59.4	47.1	0.18
Educational level (%)			0.40
- *Low*	14.5	21.6	
- *Moderate*	46.4	31.4	
- *High*	37.7	45.1	
- *‘Missing’*	1.4	2.0	
Work (%)	46.4	54.9	0.30
- *‘Missing’*	0	2.0	
Sick-leave (%)	27.5	23.5	0.46
- *‘Missing’*	0	2.0	
First visit for symptoms (%)	52.2	62.7	0.22
- *‘Missing’*	0	2.0	
Familiarity with doctor (%)			0.36
- *I don’t know the doctor*	53.6	56.9	
- *I’m familiar with the doctor*	37.7	33.3	
- *I know the doctor very well*	8.7	5.9	
- *‘Missing’*	0	3.9	

### From patients’ perspective

#### Importance and residents’ performance score of consultation aspects

Pre-intervention, the majority of patients considered affect-oriented aspects important, especially the doctor being frank towards the patient (96.9%). Chi-squared tests revealed a significant association with three consultation aspects: ‘Dr. took my problem seriously’, ‘Dr. took enough time for me’ and ‘Dr. was empathic to me’ (*p* = 0.00, *p* = 0.00 and *p* = 0.02, respectively). This implies that after residents completed the training, patients more often received what they expected. Overall, there were aspects that patients considered as not important, but that still were performed. Examples of these discrepancies are ‘Dr. examined me’ and, to a lesser extent, ‘Dr. prescribes a medicine’ and ‘Dr. refers me to other specialty’.

Post-intervention, all patients remarked the aspects ‘Dr. listened well to me’, ‘Dr. took enough time for me’ and ‘Dr. was frank to me’ as important. It is worth noticing that all affect-oriented consultation aspects were considered as performed and thereby following patients’ wish. A chi-squared test showed a significant association with ‘Dr. prescribes a medicine’ (*p* = 0.01). Thus, in this case, more patients reported to have not received a prescription for medication, independent of patients’ expectations prior to the consultation. Finally, discrepancies between relevance and performance especially concerned ‘Dr. examined me’, ‘Dr. takes final decision on treatment’ and ‘Dr. refers me to other specialty’.

#### State anxiety

Comparing pre- and post-intervention, there was a slight downward trend in anxiety level of patients after consultation with the resident, although this difference was not statistically significant (*p* = 0.85, see Table 3). The anxiety reduction during consultation was also not significantly different between the two groups.

**Table 3:** Patients’ state anxiety levels, pre- and post-intervention

	Pre-intervention	Post-intervention	*p* value
Mean anxiety score after consultation	3.35	3.33	0.85
Anxiety reduction during consultation	−0.30	− 0.29	0.72

### Residents’ empathy and relational skills

Overall, residents showed significant improvement on patient ratings of physician empathy (CARE questionnaire) (*p* = 0.04). Further evaluation of the CARE scores revealed significantly higher scores after the training on the items ‘Making you feel at ease’, ‘Really listening’ and ‘Being positive’.

The percentage of patient ratings that were ‘perfect’ (i.e., highest rating on all 10 items of the CARE), was also examined. Of the 48 patients seen by a resident before the training, 7 interactions were given a perfect score. Post-intervention, of the 43 patients, 11 interactions were given a perfect score. While not statistically significant, trends were consistent with the results described above: the percentage of perfect CARE ratings rose from 14.6% before to 25.6% after the training (see Table 4).

**Table 4:** Empathy scores of residents according to patients (measured with the CARE), pre- and post-intervention

*How was the doctor at…*	Pre-intervention	Post-intervention	*p* value
Making you feel at ease	3.89	4.30	0.02*
Letting you tell your story	4.04	4.24	0.17
Really listening	3.94	4.31	0.03*
Being interested in you as a whole person	3.82	4.20	0.07
Fully understanding your concerns	3.92	4.23	0.08
Showing care and compassion	3.88	4.19	0.09
Being positive	3.94	4.30	0.03*
Explaining things clearly	4.17	4.44	0.10
Helping you take control	3.74	4.12	0.06
Making a plan of action with you	3.98	4.31	0.10
Total CARE score	39.50	42.69	0.04*

* *p* < 0.05

### From observers’ perspective

#### Communication skills

Residents’ communication skills did not change significantly after the training. Half of the scores changed upwards and half of the scores changed downwards. However, we found a significant increase on the item ‘diagnosis’ (*p* = 0.02, see Table 5). In addition, we found an increase in residents’ empathy level, although not significant. Patient characteristics did not influence the outcomes.

**Table 5:** Resident’s communication skills measured with the MAAS-Global, pre- and post-intervention

	Pre-intervention	Post-intervention	*p* value
Introduction	2.26	2.35	0.68
Follow-up consultation	2.42	2.27	0.64
Request for help	0.83	0.83	1.00
Physical examination	4.21	4.14	0.67
Diagnosis	2.97	3.48	0.02*
Management	3.58	3.76	0.34
Evaluation of consultation	1.45	1.38	0.75
Exploration	2.25	2.15	0.51
Emotions	2.04	2.28	0.32
Information giving	3.31	3.35	0.80
Summarizations	1.49	1.20	0.34
Structuring	2.53	2.40	0.54
Empathy	3.75	3.95	0.13
Total MAAS-Global score	2.48	2.56	0.38

*p < 0.05

Inter-rater agreement was found low with an average Kappa score of 0.26 (range − 0.11 – 0.63). Therefore, the results have to be interpreted with caution.

#### Agenda setting

Agenda setting in the beginning of the consultation is a limited used method by our residents. Before the intervention 13.5% of the consultations were started with discussing the agenda setting of that consultation. After the intervention 4.9% of the consultations started with an agenda setting. This decrease in percentage is not significant (*p* = 0.29).

#### Computer use

Intrusion of computer use by the resident improved significantly from 2.35 before the intervention to 1.70 after the intervention (*p* = 0.004). This means that more residents did not use their computer during the consultation or for supportive activities only. We also examined the percentage of consultations in which this ‘perfect’ score (i.e., an intrusion score of zero or one, which means there was no computer use or for supportive activities only) was achieved. This non-significant value rose from 36.5% before the training to 56.1% after the training (*p* = 0.13).

Inter-rater agreement for intrusion of computer use, based on scores in 10 % of the videos (*n* = 11), was found sufficiently high (Kappa = 0.75).

### From residents’ perspective

The residents (*n* = 8) evaluated the training during an in-depth interview. Three main topics were derived from the interviews concerning the training: (1) positive points, (2) improvement points and (3) the future of the training and education for residents.

#### Positive points

“*The video recordings were the best part of the training, both watching your own recordings (with each other) and the personal feedback”*, according to the residents. The scientific depth of the training was also appreciated by the residents. Furthermore, residents indicated that the teachers of the training were very passionate and involved. The same applies for the appreciation for the participants from different disciplines. *“That you are (in a training) with people from different disciplines, cutting and non-cutting specialties and also GPs and a specialist as supervisors, and learn from that”.* Half of the residents were positive about practicing with actors.

#### Improvement points

Residents mentioned that the video recordings could be used more efficiently. Also the timing of the individual feedback could be changed. *“We received individual feedback on our video recorded consultations, very valuable, but I would do that before the training starts, that will increase the learning curve”.* The opinions about practicing with actors were divided. Residents mentioned that the actors’ performances were not realistic or that the residents themselves did not open up to practicing with actors. Residents also indicated that the scientific presentations from experts were interesting, but that they also lacked depth and there was repetition between the presentations and literature.

#### Future of the training and education for residents

Residents see a future for the training ‘patient-centred communication and empathy’. Only one resident preferred intervision meetings with its own colleagues over the training. The residents would like to make the future training more personal and practice oriented, with emphasis on viewing and discussing video recordings, both in the group and individually. The presentations could be connected to the topic of the day. “*For example, one day focus on empathy and another day focus on time management”.* A future training should also include residents from different specialties. Residents would structurally embed the training in the residents’ education program and feel that: *“You never stop learning, should always keep reflecting on your own communication”* and *“I think that every resident, and specialist, should receive structural communication training, whether or not combined with intervision”.*

## Discussion

The aim of this study was to evaluate a residents’ three-day training program on patient-centred communication and empathy, from the perspective of patients, observers and residents. This small scale study showed promising results. First, we found significant improvements in trained residents’ empathy and relational skills from patients’ perspective. Previous studies, also using the CARE questionnaire, showed similar outcomes [15, 31]. For example, Riess and colleagues [15] found a significant improvement in total CARE-scores in a larger group of trained residents (*n* = 99). These residents received a training including three 60-min modules spaced over 4 weeks. The results of our and other studies indicate that a training in patient-centred communication and empathy can reverse the documented decline in empathy during residency [13, 14].

In addition, our study demonstrated that most patients consider affect-oriented communication aspects the most important, especially to be treated by a frank doctor. Expectations of patients were equal in the pre- and post-intervention group. Fulfilment of these expectations are different, although this was not statistically significant. The residents more often diagnosed and explained what’s wrong and more often gave advice on what to do. Indicating that after residents completed the training in patient-centred communication, patients more often experienced to receive what they expected. These findings are in line with an earlier European study, showing that patients’ expectations for a consultation aspect are often performed, but not inevitably [32].

Furthermore, we found a non-significant anxiety reduction in patients after the intervention (*p* = 0.85). This anxiety reduction is in line with previous studies. Fogarty et al. found that physician compassion positively influences patients’ anxiety level [33]. Furthermore, an experimental study showed that the combination of an empathic communication style and raising positive expectations leads to less anxiety after the consultation [34]. Another experimental study showed that physicians’ affective communication can temper patients’ anxiety and uncertainty during bad news consultations, and enhance their ability to recall medical information [35].

Next, patient-centred communication and empathy by residents was measured from the observers’ perspective. Therefore, we used the MAAS-Global observation protocol [21]. The communication skills of trained residents did not improve significantly; half of the scores changed upwards and half of the scores downwards. However, statistical significant improvement was found for the item ‘diagnosis’, indicating better developed skills in the field of mentioning findings, causes or relations for these findings and mentioning prognosis or course of illness. Additionally, an improvement in empathy was seen. Despite the lack of significance, this outcome is important as empathy was one of the major themes in this study. We expected to find a larger improvement in MAAS-Global scores, nevertheless these outcomes are not surprisingly as the study population is small. In earlier research, Hobma et al. [17] found a score of 3.17 as the level set for the MAAS-Global scores to represent ‘adequate general practitioner communication behaviour’. We did not reach this level for mean MAAS-Global scores, but we did on the items: ‘physical examination’, ‘diagnosis’, ‘management’, ‘information giving’ and ‘empathy’. Hobma and colleagues [17] demonstrated a moderate to large effect on the communication behaviour of GPs, based on significant improvement of mean MAAS-Global scores after a training intervention consisting of assessments, selection of global topics for improvement and revalidation activities. Another study [18] investigated the effect of video feedback on practice nurses’ generic communication skills and found a statistical significant improvement in three of thirteen items on the MAAS-Global scoring list. Both studies were not performed with residents, but with GPs and practice nurses respectively, subsequently outcomes are not comparable.

In addition, our study demonstrated a significant decline in intrusion of computer use by trained residents. Previous studies showed that computer use negatively changed the proportion of time a physician looks at a patient and the amount of information given by physicians during consultations [29, 36]. Therefore, improving the computer use by residents is a substantial aspect of improving communication and empathy. It is interesting that residents’ empathy and communication significantly improves from the patients’ perspective and that from the observers’ perspective a significant improvement is found for computer use. Future research could investigate if computer use is the main point on which patients judge the communication and emphatic skills of residents.

In contrast to our expectations, agenda setting by residents did not improve after the training and even showed a non-significant decline. The percentages of consultations found in which an agenda setting was given (13.5% before and 4.9% after the intervention, respectively) appeared to be lower than the relatively low frequencies of 23 and 28% in other studies [37, 38]. Although, these studies were conducted with GPs instead of residents. Moreover, Marvel and colleagues found that trained physicians solicited the patient’s agenda more frequently than physicians without advanced training in counselling and communication skills [37]. For the future training more attention for residents’ agenda setting is warranted.

Finally, residents were positive about the training and most valued the self-reflection and feedback on the video recorded consultations. Residents would structurally embed the future training in the residents’ education program, after making some small adaptations i.e. making the training more personal and practice oriented with an emphasis on discussing video recorded consultations. Previous research found that ‘residents perceive encouragement to deliberately practice in an environment in which the value of communication skills is recognized and support is institutionalized with appropriate feedback from role models as the most important enhancing factors in communication skills learning’ [19]. This study also recommends, if it is used continuously, an approach that combines self-directed learning with observation and discussion of resident-patient consultations as an effective method [19].

### Strengths and limitations

Several strengths and limitations should be mentioned.

First, the residents participated voluntarily, which could indicate that they have an above-average interest in communication and empathy. In addition, the sample size was small and the study lacked a control group, limiting the generalizability. Another limitation was the difference in post-intervention measurements; post-measurement ranged between 6 weeks to 15 weeks after the training. The mean inter-rater reliability for the MAAS-Global was low, although general ‘agreement’ between the observers were not that different (e.g. one observer scored for example a ‘4’ while the other scored a ‘5’). However, results concerning the MAAS-Global need to be interpreted with caution. Furthermore, observers were not blinded to scoring consultations belonging to the pre- or post-intervention group, which could cause bias. Although, there were no major improvements in communication skills of residents observed which demonstrates bias was probably not present.

In addition, the difference between empathy, sympathy and compassion is difficult to distinguish (e.g. [39]) Although, our study focused on emphatic behaviour of the residents they could have shown sympathy or compassioned behaviour instead or as well.

Despite the small sample size, this pilot study suggests that empathic and communicative skills can be taught to resident physicians and results in several significant improvements. In addition, evaluation of the training was measured from three perspectives; patients, observers and residents. This strengthens the reliability and objectivity of the results. Besides, six experts derived from multiple specialties trained the residents and residents were also derived from several specialties. Finally, the outcomes are based on multiple video recorded consultations with different patients. The training given consisted of several teaching methods and a significant part of the training was reserved for feedback on residents’ actual performance. The latter has proven to be the best way to teach residents [40, 41].

### Implications for future research and clinical practice

This small scale study showed a significant improvement on residents’ empathy and computer skills after the training ‘patient-centred communication and empathy’. For future research, a larger sample size, including residents from more specialties, is needed to show if our results are still valid and to examine the sustainability of the learned skills. Based on previous research in this area [41, 42], we expect that video feedback needs to be conducted on a regular basis for residents to maintain the learned skills, and also become part of a systematic training program.

In addition, it would be interesting to take residents’ specialty, gender, age and years of experience into account. The vitality of residents could be another interesting topic for future research, since previous studies found a decrease in distress and burn-outs among physicians who conducted empathic consultations [11, 12, 43]. To define the clinical relevance, it is necessary to conduct a study to determine the minimal improvement on each scoring list to be clinically relevant.

The training itself could also be improved. First of all by emphasizing the importance of agenda setting and how to implement this in a consultation. The residents themselves would like to make the future training more personal and practice oriented, with emphasis on viewing and discussing video recordings, both in the group and individually. As mentioned before, a previous study among residents also recommends an approach that combines self-directed learning with observation and discussion of resident-patient consultations as an effective method [19].

## Conclusions

A brief training ‘patient-centred communication and empathy’ significantly increased residents’ empathy scores according to patients and significantly decreased residents’ computer use according to observers. These findings indicate that the quality of patient-centred care can be improved by integrating patient-centred communication into residency programs, at an academic medical health centre. In addition, almost all residents were positive about the training, especially about the self-reflection and feedback on the video recorded consultations. However, as this was a first pilot study more evidence is needed from a larger group of residents. In addition, the training itself could be improved. Nevertheless, structurally embedding the training in the residents’ education program is recommended.

## Abbreviations

CARE: The Consultation and Relational Empathy Measure
GP: General Practitioner
MAAS-Global: Maastrichtse Anamnese en Advies Scorelijst
QUOTE COMM: The Quality of Communication Through Patient’ Eyes
STAI: Spielberger State-Trait Anxiety Inventory
